# On correct computation of confidence intervals for kinetic parameters

**DOI:** 10.14814/phy2.14180

**Published:** 2019-07-18

**Authors:** Maria Pia Francescato, Valentina Cettolo, Ruggero Bellio

**Affiliations:** ^1^ Department of Medicine University of Udine Udine Italy; ^2^ Department of Economics and Statistics University of Udine Udine Italy

Two papers have been recently published in Physiology Reports (Goulding et al. 2018a,b) that compare the kinetic parameters of three physiological signals during transitions in exercise intensity. The authors, using a commercial statistical software to apply the nonlinear regression technique, reported among their results also “the 95% confidence intervals (CIs) for the derived parameter estimates”. The investigated signals (oxygen uptake, heart rate, and NIRS data) were acquired with different time resolutions, that is breath‐by‐breath, at 1  (1 Hz) and 0.5  (2 Hz), respectively. Before running the nonlinear regression, the oxygen uptake data only underwent an interpolation procedure to produce second‐by‐second values (i.e. at 1 Hz). It should be noted, however, that this procedure was not even supported by the results reported by Benson et al. (2017), although it was suggested in their abstract (Francescato et al. 2017). In fact, this procedure does not add new information to the data, rather it reiterates the already available information in the newly introduced data points, invalidating the CIs obtained by the calculations (Francescato et al. 2015).

For each of the estimated parameters, the statistical packages provide directly the 95% Confidence Limits and/or provide the Asymptotic Standard Error (ASE); commonly used confidence limits and ASE are related to each other, as follows:

$$\begin{array}{c} \text{Lower confidence limit}=\text{Estimated value}-(1.96\times \text{ASE})\\ \text{Upper confidence limit}=\text{Estimated value}+(1.96\times \text{ASE}) \end{array}$$

where the constant 1.96 is valid under the hypothesis that the number of degrees of freedom is greater than 30, thus the above equations hold true for the majority of the reported physiological phenomena (Bates and Watts 1988).

The ASE is calculated on the basis of the variance‐covariance matrix and is only an estimate of the uncertainty; independently of the information carried by each of the considered data points, the greater is their number, the smaller the ASE will become (Francescato et al. 2014a). By applying the interpolation procedure on the oxygen uptake data, the authors considered an artificially higher number of data points, that carried “cloned” information; consequently, the obtained and reported CIs were falsely smaller. Conversely, the CIs reported for the NIRS and heart rate data were appropriate, since the native time resolution of these parameters is finest compared to the data acquisition one, and the data points were not “cloned”.

Notably, the statistical packages running the nonlinear regression are able to deal with data showing a variable time resolution, without the need of data reordered in time. As a matter of fact, it has been shown that, simply appending one after the other, the gas exchange data of the repeated transitions (“stacking”), without modifying the native time resolution, allow to obtain ASE values for the time parameters (time delay and time constant) that yield an appropriate quantification of uncertainty (Francescato et al. 2014b). We believe that, to retain all the information contained in values collected with a time resolution that changes throughout the acquisition, the “stacking” procedure is the most correct one.
